# Nitrotyrosine Level Was Associated with Mortality in Patients with Acute Kidney Injury

**DOI:** 10.1371/journal.pone.0079962

**Published:** 2013-11-20

**Authors:** Jing Qian, Huaizhou You, Qiuyu Zhu, Shuai Ma, Ying Zhou, Ying Zheng, Junfeng Liu, Dingwei Kuang, Yong Gu, Chuanming Hao, Feng Ding

**Affiliations:** Division of Nephrology, Huashan Hospital, Fudan University, Shanghai, China; Universidade de Sao Paulo, Brazil

## Abstract

**Background:**

To examine the characteristics of oxidative stress in patients with acute kidney injury (AKI) and investigate the association between plasma nitrotyrosine levels and 90-day mortality in patients with AKI.

**Methodology/Principal Findings:**

158 patients with hospital-acquired AKI were recruited to this prospective cohort study according to RIFLE (Risk, Injury, Failure, Lost or End Stage Kidney) criteria. Twelve critically ill patients without AKI and 15 age and gender-matched healthy subjects served as control. Plasma 3-nitrotyrosine was analyzed in relation to 90-day all cause mortality of patients with AKI. The patients with AKI were followed up for 90 days and grouped according to median plasma 3-nitrotyrosine concentrations. Highest 3-NT/Tyr was detected in patients with AKI compared with healthy subjects, and critically ill patients without AKI (ANOVA *p*<0.001). The 90-day survival curves of patients with high 3-NT/Tyr showed significant differences compared with the curves of individuals with low 3-NT/Tyr (*p* = 0.001 by log rank test). Multivariate analysis (Cox regression) revealed that 3-NT/Tyr (*p* = 0.025) was independently associated with mortality after adjustment for age, gender, sepsis and Acute Physiology and Chronic Health Evaluation (APACHE) II score.

**Conclusions/Significance:**

There is excess plasma protein oxidation in patients with AKI, as evidenced by increased nitrotyrosine content. 3-NT/Tyr level was associated with mortality of AKI patients independent of the severity of illness.

## Background

In normal physiologic conditions, a homeostatic balance exists between the formation of reactive oxygen species (ROS) and their removal by endogenous antioxidant compounds [1]. Oxidative stress occurs when this balance is disrupted by excessive production of ROS and/or by inadequate antioxidant defenses [2]. Both of these imbalances can occur in acute kidney injury (AKI). Indeed, renal failure itself is now recognized as an additional stimulus for increased oxidative stress [3], [4]. Furthermore, an increase in oxidative stress is considered an important pathogenic mechanism in the development of ischemic and toxic renal tubular injury [5]–[9].

Up to now, no agreement has been reached as to the cellular sources of radicals in AKI: oxidation of hypoxanthine [10], mitochondrial production of free radicals, and lipoxygenase-or prostaglandin H-dependent production during arachidonic acid metabolism [11]. It has been appreciated that hydroxyl radical-like activities are generated from peroxynitrite [12]. This latter compound is emerging as one of the important sequelae of oxidative and nitrosative stress: the reaction between superoxide ion and nitric oxide (NO) proceeds at a near-diffusion-limited rate, thus resulting in an almost instantaneous generation of peroxynitrite in preference to nitrites, nitrates, or hydrogen peroxide [12]. Peroxynitrite is a potent oxidant that promotes nitration of protein tyrosine residues producing a distinctive “molecular fingerprint” for nitric oxide–derived oxidants, nitrotyrosine [13], [14]. The ready accessibility of plasma proteins for sampling, the relatively long plasma half-lives of many proteins, and the well-characterized biochemical pathways of protein and amino acid oxidation make plasma protein oxidation an attractive *in vivo* biomarker of oxidative reactions [15]–[17].

Few clinical studies have examined the prevalence of oxidative stress in patients with AKI, and none has examined whether oxidative stress is associated with adverse clinical outcomes. In this study, we compared measurements of plasma nitrotyrosine in patients with AKI, healthy subjects, critically ill patients without AKI, and investigated whether plasma protein oxidation levels were associated with 90-day mortality.

## Materials and Methods

### Selection of Participants

#### Patients with AKI

We prospectively studied a consecutive cohort of 158 adult patients with hospital-acquired AKI from February 2009 to February 2010 at Huashan Hospital, a tertiary hospital affiliated to Fudan University with 30 wards comprising 1500 beds in Shanghai, China. Eligible patients were ≥18 years old and diagnosed with AKI during hospitalization. Exclusion criteria included 1) confirmed and/or suspected acute glomerulonephritis, acute interstitial nephritis, renal vasculitis or postrenal etiology for AKI; 2) diagnosed as metastasis tumors; 3) admission with AKI; 4) unknown premorbid increased serum creatinine; 5) refused to be enrolled into our study; 6) enrollment in other studies; 7) pregnancy; 8) use of anti-oxidant. All patients were followed up for 90 days after the diagnosis of AKI. The primary outcome was all-cause mortality. The survival of the patients was confirmed by the National Population Information Query System.

#### Critically ill patients without AKI

Twelve critically ill patients without AKI from Huashan Hospital during the same period served as control subjects. The absence of AKI was determined by the serum creatinine level. All patients were matched according to age, gender and Acute Physiology and Chronic Health Evaluation (APACHE) II score with AKI patients.

#### Healthy subjects

A group of 15 age- and gender-matched healthy subjects were enrolled as control. Healthy subjects were randomly obtained from the healthy examinators of healthcare center of Huashan Hospital during the same period.

None of the participants were taking drugs known to interfere with the anti-oxidant levels during the 90-day follow-up, including vitamin C and vitamin E. Patients with AKI and critically ill patients without AKI remained under the care of the hospital unit to which they were admitted. The study investigators did not participate in the patients’ medical care unless invited. The study was approved by the ethics committee of Huashan Hospital, Fudan University (approval number: 2009-097). All patients gave written informed consent, and the Declaration of Helsinki was adhered to.

### Study Definitions

AKI was defined using the Risk, Injury, Failure, Loss, and End-stage (RIFLE) classification criteria. We classified patients according to the RIFLE class (class Risk, class Injury, or class Failure) when AKI was first diagnosed, defined as a fold change in serum creatinine from baseline serum creatinine within 1 week. RIFLE class was defined as Risk (fold change ≥1.5), Injury (fold change ≥2.0), or Failure (fold change ≥3.0). Baseline serum creatinine was obtained in all patients from 1 week before AKI was diagnosed. According to consensus guidelines, sepsis syndrome was considered to be present in patients in whom infection was accompanied by at least 2 Systemic Inflammatory Response Syndrome (SIRS) criteria. Infection was diagnosed according to usual clinical, laboratory, and microbiological parameters. Patients with operation were defined as those who had undergone a surgical operation within 1 week before diagnosis of AKI.

### Blood Sampling

Blood samples were obtained from patients with AKI within 24 hours after AKI was diagnosed. Blood samples were obtained from critically ill patients without AKI within 48 hours after admission. Samples were obtained from healthy subjects at the time of enrollment. Blood for serum measurements was drawn into BD Vacutainer serum-separating tubes that contained a clot activator. Tubes were kept at room temperature and centrifuged within 1 hour of the blood draw. All blood samples were stored at −80°C until analysis.

### Clinical Evaluation

Baseline demographics were recorded, including age, gender, and comorbidities such as hypertension, diabetes mellitus, cardiovascular disease, chronic hepatic disease, chronic kidney disease, chronic obstructive pulmonary disease, and malignant tumors. The following data were also recorded upon patient enrollment: the possible cause of AKI, the presence of sepsis, and the need for mechanical ventilation. We further assessed Acute Physiology and Chronic Health Evaluation (APACHE) II score, and Subjective Global Assessment (SGA). All these patients were followed up for 90 days. The primary outcome was all-cause mortality.

### Laboratory Procedures

The white blood cell, neutrophilic granulocyte, and hemoglobin concentrations were measured by an automated haematology analyzer (Sysmex XE-2100). The serum levels of creatinine, alanine aminotransferase, aspartate aminotransferase, albumin, calcium and phosphate were determined by a biochemistry autoanalyzer (Hitachi 7600-020b).

### 3-nitrotyrosine (3-NT) Determination

The determination of 3-NT in plasma in protein-bound form was performed according to the methods described [18], [19] with slight modifications. In brief, 100 µl of plasma was mixed with 400 µl of HBr (2 M) prior to the precipitation with 50% trichloroacetic acid (TCA). Obtained protein pellets were then delipidated three times with H_2_O_2_/Methanol/Ethyl acetate (1∶3∶7) and hydrolyzed under nitrogen in 0.4 ml of 8 M HBr containing 1% phenol (w/v) at 115°C for 24 h. After freeze-drying under vacuum, the resulting hydrolysates were then reconstituted and stored as described above. The separations for all target indices were conducted on a Hypersil AA-ODS C18 column (5 µm, 2.1×200 mm, Agilent,USA) by mobile phase (8% methanol/40 mM sodium phosphorate/1 mM KCl, pH 2.5) at a flow rate of 0.25 ml/min. The detections were functioned by means of a UV detector at 280 nm for tyrosine molecular monitoring and an electrochemical detector (Model 1049A, Agilent, USA) at 0.90 V of applied oxidation potential for measurements of the chlorinated/nitrated tyrosines, respectively. Both detectors were connected in tandem, so that the measurements could be carried out simultaneously. Because the absolute value of 3-NT is small, and can be affected by the total tyrosine level, the 3-NT level was expressed as ratio of 3-nitrotyrosine to tyrosine (µmol·L^−1^/mmol·L^−1^).

### Statistical Analyses

Normally distributed variables are expressed as mean ± standard deviation (SD) and were compared using one-way analysis of variance (ANOVA) or the *t* test. Nonnormally distributed variables were expressed as medians with interquartile range and were compared using the rank sum test. Categorical variables are expressed as percentages and compared using Pearson’s chi-square test or Fisher’s exact test. Correlations among continuous data were performed using Pearson’s correlation coefficients. In multivariate analysis, Cox proportional hazards regression was used to identify independent predictors of mortality in AKI patients. Covariates including age, gender sepsis and APACHE II score were used for stepwise adjustment. Overall survival at 90 days between the high group and the low group which were divided according to the median of the ratio of 3-nitrotyrosine to tyrosine was evaluated using Kaplan-Meier analysis, and differences between them were tested using the log-rank test. The terminal event was death and the patients lost to follow-up were censored at their last observation. All tests were two-tailed, and statistical significance was defined as *p*<0.05. The SPSS statistical software program (version 15.0, SPSS Inc., Chicago, IL) was used for all analyses.

## Results

### Demographic and Clinical Characteristics of the AKI Study Cohort and Control Groups

A total of 263 patients were diagnosed as acute kidney injury during the one-year study period. Of these, 13 patients were excluded due to the absence of baseline serum creatinine or admission with AKI, and 38 patients were also excluded due to acute glomerulonephritis, acute interstitial nephritis, renal vasculitis or postrenal etiology for AKI. Of the 198 patients, 48 patients used anti-oxidant during the study period and 6 patients refused to be enrolled into our study. Finally, 158 patients were enrolled. Of the 158 patients studied (111 men and 47 women) with a mean age of 62.7±17.8 years who met the criteria for AKI, 86 patients (54.4%) underwent surgery and 62 patients (39.2%) developed sepsis. Upon admission, the median serum creatinine level was 0.7 mg/dL, which increased to 1.5 mg/dL when AKI was diagnosed. The mean APACHE II score was 20.2±10.0, and 88 patients (55.7%) survived longer than 90 days.

All patients with AKI were classified into three RIFLE subgroups (Risk, Injury and Failure) by the RIFLE staging criteria according to their changes in serum creatinine levels within 1 week. The sample size of each subgroup (Risk, Injury and Failure) was 74 (46.8%), 36 (22.8%) and 48 (30.4%) patients, respectively. Comparison among these three subgroups revealed no significant differences in age, gender or comorbid conditions. The percentage of patients with operations was highest in the Risk subgroup and lowest in the Failure subgroup. The lowest serum albumin and highest serum phosphate levels were found in patients with AKI, and the APACHE II scores significantly increased with the severity of AKI (*p = *0.004, *p = *0.035, and *p*<0.001, respectively).

The demographic and clinical characteristics as stratified by the RIFLE stage of AKI are shown in Table 1.

**Table 1:** Baseline demographic and clinical data of patients at the time of acute kidney injury diagnosis stratified by RIFLE stages.

	No. (%)				
Characteristic	Total	Risk	Injury	Failure	*p* value
	(n = 158)	(n = 74)	(n = 36)	(n = 48)	
Age (yr), mean (s.d.)	62.7±17.8	65.4±16.3	65.3±17.7	56.6±19.2	0.100
Gender(%female)	47(29.7)	22(29.7)	13(36.1)	12(25.0)	0.796
Baseline Scr (mg/dL)	0.7±0.3	0.7±0.2	0.7±0.2	0.6±0.3	0.368
Scr when AKI diagnosed (mg/dL)	1.5(1.2, 2.1)	1.2±0.4	1.6±0.4	3.8±3.2	<0.001
Comorbid conditions					
Hypertension (%)	73(46.2)	32(43.2)	19(52.8)	22(45.8)	0.800
CVD (%)	20(12.7)	10(13.5)	6(16.7)	4(8.3)	0.739
DM (%)	22(13.9)	12(16.2)	4(11.1)	6(12.5)	0.824
Chronic hepatic disease (%)	10(6.3)	6(8.1)	0(0)	4(8.3)	0.436
Malignant tumor (%)	16(10.1)	6(8.1)	4(11.1)	6(12.5)	0.852
COPD	6(3.8)	2(2.7)	2(5.6)	2(4.2)	0.885
CKD (%)	2(1.3)	0(0)	0(0)	2(4.2)	0.307
AKI etiology					
Ischemic (%)	60(38.0)	28(37.8)	16(44.4)	16(33.3)	0.715
Nephrotoxic (%)	49(31.0)	26(35.1)	11(30.6)	12(25.0)	0.729
Others (%)	49(31.0)	20(27.1)	9(25.0)	20(41.7)	
Operation (%)	86(54.4)	52(70.3)	18(50.0)	16(33.3)	0.012
Sepsis (%)	62(39.2)	26(35.1)	16(44.4)	20(41.7)	0.739
Mechanical ventilation (%)	64(40.5)	22(29.7)	16(44.4)	26(54.2)	0.120
MAP (mmHg)	89±17	91±14	86±17	87±22	0.580
WBC(×10^3^/µL)	13.1±6.5	12.6±6.1	15.2±6.7	12.1±6.7	0.227
Neutrophilic granulocyte (%)	79.1(76.6, 87.1)	81.2(75.1, 85.6)	85.1(77.5, 87.3)	79.9±16.2	0.305
Hemoglobin (g/dL)	10.9±2.7	11.3±2.0	10.9±2.9	10.5±3.4	0.484
Platelet (×10^3^/µL)	141.6±97.9	156.7±105.6	135.1±76.5	123.3±98.7	0.370
ALT (U/L)	32.5(20.0, 65.3)	27.5(17.5, 57.0)	38.5(20.0, 109.8)	31.0(13.0, 95.0)	0.487
AST (U/L)	41.0(25.0, 97.3)	34.5(23.5, 76.5)	64.5(29.5, 158.5)	42.0(18.0, 112.0)	0.207
Serum albumin (g/dL)	3.2±0.7	3.5±0.7	3.3±0.7	2.9±0.6	0.004
Serum total calcium (mmol/L)	2.1±0.3	2.1±0.2	2.1±0.3	2.0±0.3	0.252
Serum phosphate (mmol/L)	1.3±0.7	1.1±0.6	1.4±0.8	1.7±0.8	0.035
Cholesterol (mmol/L)	3.7±1.5	3.3±1.4	3.4±1.8	3.5±1.3	0.791
Prealbumin (mg/dL)	14.1±6.0	14.8±6.2	14.4±6.8	12.7±4.7	0.372
CRP (mg/dL)	8.8±6.8	7.0±6.6	9.9±7.2	11.0±6.5	0.060
APACHE II	20.2±10.0	15.5±8.8	22.7±7.8	25.6±9.9	<0.001

**Note:** Data were obtained at the time of AKI diagnosis, unless otherwise noted.

**Abbreviations:** RIFLE, Risk, Injury, Failure, Loss, or End-stage kidney disease staging criteria; CVD, cardiovascular diseases; DM, diabetes mellitus; COPD, chronic obstructive pulmonary disease; CKD, chronic kidney disease; Scr, serum creatinine; AKI, acute kidney disease; MAP, mean artery pressure; WBC, white blood cell; ALT, Alanine aminotransferase; AST, Aspartate aminotransferase; CRP, C- reactive protein; APACHE II, Acute Physiology and Chronic Health Evaluation II.

Fifteen healthy subjects and 12 critically ill patients without AKI served as control. The mean age of healthy subjects and critically ill patients without AKI was 58.3±18.1 and 56.1±17.0 years, and the proportion of female was 33.3% and 25.0%, respectively. No statistical difference was found in age or gender among the control groups and AKI cohort (*p*>0.05). In addition, the APACHE II score of critically ill patients without AKI was matched with the patients with AKI (*p*>0.05).

### Ratio of 3-nitrotyrosine to tyrosine (3-NT/Tyr) in Healthy Subjects, Critically ill Patients without AKI, and Patients with AKI

**Figure 1:**
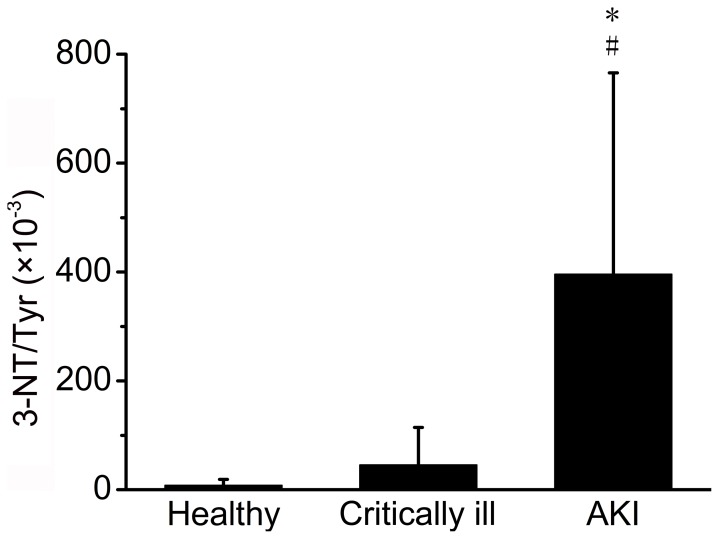
3-NT/Tyr among healthy subjects, critically ill patients without AKI and patients with AKI. * t test *p*<0.001, AKI patients compared to criticall ill patients; # t test *p*<0.001, AKI patients compared to healthy subjects.

Figure 1 shows the ratio of 3-nitrotyrosine to tyrosine (3-NT/Tyr) among healthy subjects, critically ill patients without AKI and patients with AKI. Highest 3-NT/Tyr (395.9±369.8×10^−3^) was detected in patients with AKI compared with healthy subjects (8.1±10.7×10^−3^), and critically ill patients without AKI (45.9±68.4×10^−3^). Significant difference was found in 3-NT/Tyr between AKI patients and healthy subjects (t test, *p*<0.001), and also between AKI patients and critically ill patients without AKI (t test, *p*<0.001).

### 3-NT/Tyr in Patients with Different RIFLE Stages of AKI

**Figure 2:**
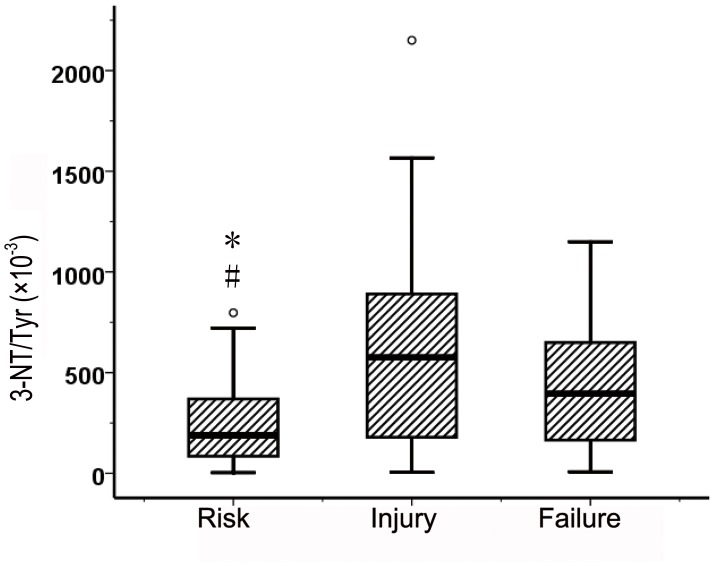
3-NT/Tyr in 158 patients with AKI stratified by RIFLE stages. * t test *p* = 0.002, AKI patients in risk stage compared to in injury stage; # t test *p* = 0.007, AKI patients in risk stage compared to in failure stage.

The levels of 3-NT/Tyr stratified by the RIFLE criteria were 240.7±195.6, 663.3±528.5, 433.4±316.4 (×10^−3^). Significant difference was found in 3-NT/Tyr between AKI patients in risk stage and in injury stage (t test, *p* = 0.002), and also between AKI patients in risk stage and in failure stage (t test, *p* = 0.007) (Figure 2).

### Correlation Analysis of 3-NT/Tyr with APACHE II Score and Serum Creatinine

**Figure 3:**
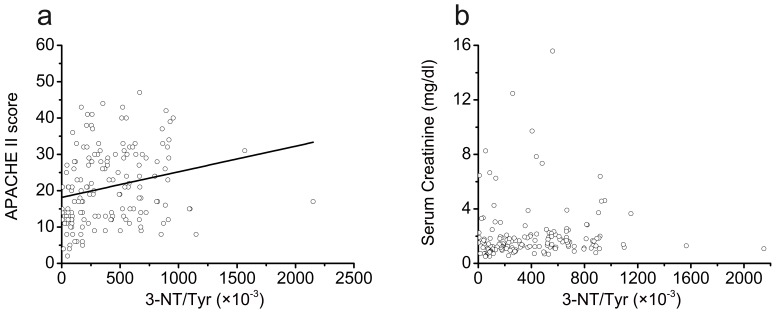
Correlations analysis of 3-NT/Tyr with APACHE II score and serum creatinine as AKI diagnosed in 158 patients with AKI. (a) correlation between 3-nitrotyrosine to tyrosine and APACHE II score, adjusted r^2^ = 0.048, *p* = 0.003. (b) correlation between 3-nitrotyrosine to tyrosine and serum creatinine as AKI diagnosed, *p* = 0.544. APACHE II, Acute Physiology and Chronic Health Evaluation II.

The correlation analysis indicated that ratio of 3-NT/Tyr had a close positive correlation with the APACHE II score, and the correlation was statistically significant (adjusted r^2^ = 0.048, *p* = 0.003) (Figure 3a), but it has no correlation with serum creatinine (Figure 3b).

### Mortality Predictability of 3-NT/Tyr during a 90-Day Period

We further used the median value of 3-NT/Tyr as cut-off point to divide the158 patients with AKI into two subgroups: low 3-NT/Tyr (<296×10^−3^) and high 3-NT/Tyr (>296×10^−3^).

**Figure 4:**
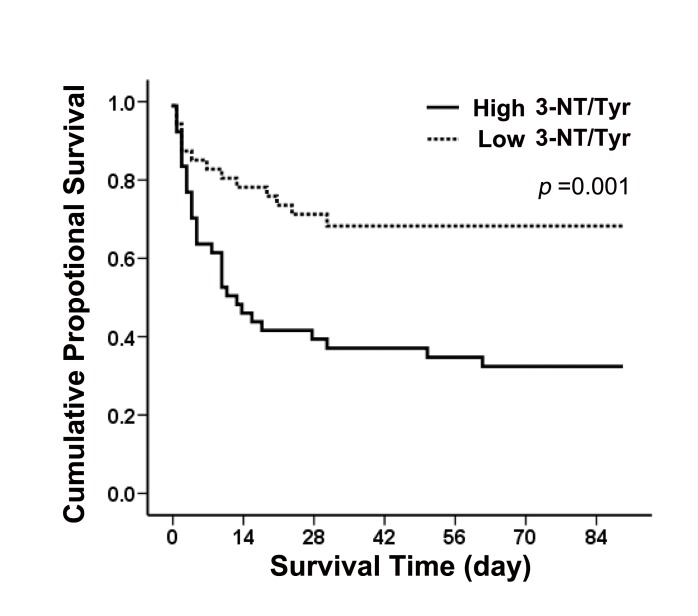
Kaplan-Meier analysis for the cumulative percentage of surviving patients at 90 days according to different 3-NT/Tyr. Kaplan-Meier plots for probability of survival at 90 days between high and low 3-NT/Tyr. Significant difference between the two subgroups was found (log rank *p* = 0.001).

Among patients in the high 3-NT/Tyr level group, 68.4% died, compared with 29.1% in the low 3-NT/Tyr level group. According to the Kaplan-Meier plot, the survival curves of those patients with high 3-NT/Tyr show significant difference with those with low 3-NT/Tyr. At 90 days after patient enrollment, the mortality rate was significantly higher in the group with high 3-NT/Tyr (*p* = 0.001 by the log rank test) (Figure 4).

**Figure 5:**
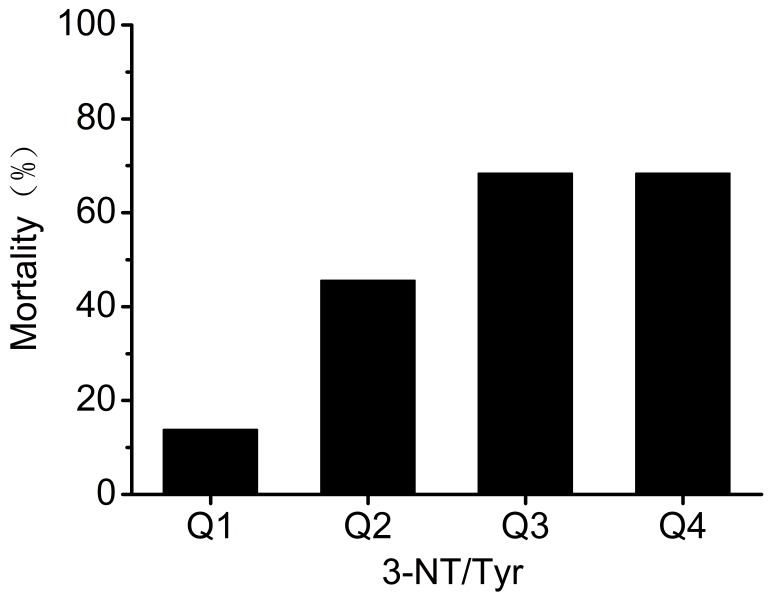
90-day overall mortality according to quartiles of 3-NT/Tyr (χ^2^ = 17.6, *p* = 0.001).

The 90-day overall mortality was 13.6%, 45.55%, 68.2% and 68.2%, respectively in quartiles of 3-NT/Tyr (Figure 5). A rising trend in 90-day overall mortality was found with the increase of 3-NT/Tyr (χ^2^ = 17.6, *p* = 0.001).

In multivariable analysis of these selected possible predictors for mortality of AKI patients, we controlled the demographic factors including age and gender as model 1, controlled age, gender and APACHE II score as model 2, controlled age, gender and sepsis as model 3, controlled age, gender, APACHE II score and sepsis as model 4. Table 2 showed that when adjusted for age, gender, sepsis and the severity of illness (APACHE II score), 3-NT/Tyr remained an independent predictor of 90-day overall mortality (3-NT/Tyr: *p* = 0.025).

**Table 2:** Multivariate Cox regression analysis for 3-NT/Tyr and other selected possible predictors of mortality in patients with AKI.

	3-NT/Tyr (×10^−3^)		Gender		Age		Sepsis		APACHE II	
	HR(95% CI)	*p*	HR(95% CI)	*p*	HR(95% CI)	*p*	HR(95% CI)	*p*	HR(95% CI)	*p*
Model 1	1.001(1.000,1.002)	0.001	1.217(0.642,2.305)	0.547	1.002(0.985,1.019)	0.823	/	/	/	/
Model 2	1.001(1.000,1.002)	0.018	2.140(1.053,4.347)	0.035	1.003(0.986,1.020)	0.727	/	/	1.163(1.1141.213)	<0.001
Model 3	1.001(1.000,1.002)	0.001	1.259(0.658,2.406)	0.486	1.001(0.984,1.018)	0.936	1.093(0.583,2.049)	0.781	/	/
Model 4	1.001(1.000,1.002)	0.025	2.222(1.087,4.545)	0.029	1.008(0.989,1.026)	0.417	0.490(0.249,0.965)	0.039	1.181(1.128,1.236)	<0.001

**Note:** the multivariate analysis sequentially adjusted models for covariates as follows: Model 1, adjusted for age, gender; Model 2, adjusted for age, gender and APACHE II score; Model 3, adjusted for age, gender and sepsis; Model 4, adjusted for age, gender, APACHE II score and sepsis.

## Discussion

Our study is the first, to our knowledge, to demonstrate that there is excess plasma protein oxidation and nitration in patients with AKI, as evidenced by increased nitrotyrosine content. 3-nitrotyrosine (3-NT), a marker specific for protein modification by nitric oxide–derived oxidants may serve as a novel predictor for 90-day overall mortality.

3-nitrotyrosine is a stable marker of generation of peroxynitrite (ONOO^−^), a highly reactive oxidative and nitrosative species which derives from the *in vivo* reaction of superoxide anion and nitric oxide (NO) [9]. The paramagnetic NO rapidly reacts with superoxide to form peroxynitrite anion, a potent nitrating and oxidizing agent, resulting in oxidative damage to proteins, lipids, carbohydrates, DNA, and sulfhydryl groups. ONOO^−^ can nitrate aromatic amino acids, and nitration on the three position of tyrosine can form free or protein associated nitrotyrosine [20]–[22]. Nitrotyrosine has been used as a molecular footprint of ONOO^−^ formation. This now-established posttranslational modification attracts considerable interest to biomedical research, because it can alter protein function, is associated to acute and chronic disease states such as inflammation, cardiovascular disease, neurodegeneration, diabetes, and other pathologies, and can be a predictor of disease risk.

In animal models of acute kidney injury, it has been demonstrated that ischemic and endotoxin induced renal injury are accompanied by nitrotyrosine formation in the renal tissues [23], [24]. Up to now, there was no report of nitrotyrosine expression in patients with AKI. Our study firstly demonstrated that the systemic levels of 3-NT were significantly increased in AKI patients. 3-NT levels became elevated as APACHE II scores rose. More importantly, systemic levels of protein-bound 3-NT were associated with the 90-day overall mortality even following multivariable adjustments for APACHE II score and sepsis. Taken together, these results suggest that nitrotyrosine measurements may prove useful both in assessing AKI severity and predicting the mortality of AKI patients.

The results of our study suggested that in patients with AKI, the pathological conditions such as ischemia might induce iNOS-derived NO production. NO would react with increased ROS, resulting in increased peroxynitrite formation and further nitrotyrosine formation. Our study found high 3-NT expressions in patients’ plasma in addition to in animal renal tissues. Peroxynitrite is thought to be, at least in part, responsible for renal damage, due to peroxynitrite associated oxidative and nitrosative effects on several targets, such as sulfydryl groups and aromatic rings of proteins, cell membrane lipids and nucleic acid [9]. It has been confirmed by a series of studies showing that strategies directly preventing peroxynitrite generation or accelerating its degradation prevented the generation of nitrotyrosine in injured tubular cells, ameliorated renal histological damage, and reduced functional renal alterations in kidney injury. So nitrothrosine, footprint of peroxynitrite formation, may play a role in predicting the mortality of AKI patients.

There were some limitations in our study. Firstly, this was an observational, single-center and relatively small size study. Secondly, the studied population was composed by heterogeneous AKI patients in a tertiary comprehensive hospital. Selection bias may exist. Thirdly, most of the data were obtained at a single time point, whereas the clinical course of development and manifestations of AKI in critically ill patients is heterogeneous and varies over time. In conclusion, patients with AKI manifest a marked increase nitrotyrosine formation. Elevated nitrotyrosine levels were associated with overall mortality. These data suggest that increased oxidative stress may be a potential target for future pharmacologic and therapeutic applications. Further evaluation of nitrotyrosine levels as predictors of mortality and as means of monitoring risk reduction attendant with antioxidant therapy are warranted.

There is excess plasma protein oxidation in patients with AKI, as evidenced by increased nitrotyrosine content. 3-NT was associated with mortality of AKI patients independent of the severity of illness.

## References

[1] Gutteridge J (1999) Mitchell (1999) Redox imbalance in the critically ill. Br Med Bull 55: 49–75. 10695079 10.1258/0007142991902295

[2] Macdonald J, Galley H, Webster N (2003) Oxidative stress and gene expression in sepsis. Br J Anaesth 90: 221–232. 12538380 10.1093/bja/aeg034

[3] Himmelfarb J, Ikizler TA, Stenvinkel P, Hakim RM (2002) The elephant in uremia: Reflections on oxidant stress as a unifying concept of cardiovascular disease in uremia. Kidney Int 62: 1524–1538. 12371953 10.1046/j.1523-1755.2002.00600.x

[4] Descamps-Latscha B, Drueke T, Witko-Sarsat V (2001) Dialysis-induced oxidative stress: Biological aspects, clinical consequences, and therapy. Semin Dial 14: 193–199. 11422926 10.1046/j.1525-139x.2001.00052.x

[5] Paller MS, Hoidal JR, Ferris TF (1984) Oxygen free radicals in ischemic acute renal failure in the rat. J Clin Invest 74: 1156–1164. 6434591 10.1172/JCI111524 PMC425281

[6] Zager RA (1997) Pathogenetic mechanisms in nephrotoxic acute renal failure. Semin Nephrol 17: 3–14. 9000545

[7] Baliga R, Ueda N, Walker PD, Shah SV (1997) Oxidant mechanisms in toxic acute renal failure. Am J Kidney Dis 29: 465–477. 9041227 10.1016/s0272-6386(97)90212-2

[8] Baliga R, Ueda N, Walker PD, Shah SV (1999) Oxidant mechanisms in toxic acute renal failure. Drug Metab Rev 31: 971–997. 10575556 10.1081/dmr-100101947

[9] Noiri E, Nakao A, Uchidna K, Tsukahara H, Ohno M, et al (2001) Oxidative and nitrosative stress in acute renal ischemia. Am J Physiol 281: 948–957. 10.1152/ajprenal.2001.281.5.F948 11592952

[10] Osswald H, Schmitz HJ, Kemper R (1977) Tissue content of adenosine, inosine and hypoxanthine in the rat kidney after ischemia and postischemic recirculation. Pflugers Arch 371: 45–49. 563575 10.1007/BF00580771

[11] Bonventre JC (1993) Mechanism of ischemic acute renal failure. Kidney Int 43: 1160–1178. 8510397 10.1038/ki.1993.163

[12] Beckman JS, Beckman TW, Chen J, Marshall PA, Freeman BA (1990) Apparent hydroxyl radical production by peroxynitrite: implications for endothelial injury from nitric oxide and superoxide. Proc Natl Acad Sci USA 87: 1620–1624. 2154753 10.1073/pnas.87.4.1620 PMC53527

[13] Beckman JS, Koppenol WH (1996) Nitric oxide, superoxide, and peroxynitrite: the good, the bad, and ugly. Am J Physiol 271: 1424–1437. 10.1152/ajpcell.1996.271.5.C1424 8944624

[14] Hazen SL, Zhang R, Shen Z, Wu W, Podrez EA, et al (1999) Formation of nitric oxide-derived oxidants by myeloperoxidase in monocytes: pathways for monocyte-mediated protein nitration and lipid peroxidation in vivo. Circ Res 85: 950–958. 10559142 10.1161/01.res.85.10.950

[15] Davies MJ, Fu S, Wang H, Dean RT (1999) Stable markers of oxidant damage to proteins and their application in the study of human disease. Free Radic Biol Med 27: 1151–1163. 10641706 10.1016/s0891-5849(99)00206-3

[16] Heinecke JW (2002) Oxidized amino acids: Culprits in human atherosclerosis and indicators of oxidative stress. Free Radic Biol Med 32: 1090–1101. 12031894 10.1016/s0891-5849(02)00792-x

[17] Sohal RS (2002) Role of oxidative stress and protein oxidation in the aging process. Free Radic Biol Med 33: 37–44. 12086680 10.1016/s0891-5849(02)00856-0

[18] Zhang WZ, Lang C, Kaye DM (2007) Determination of plasma free 3-nitrotyrosine and tyrosine by reversed-phase liquid chromatography with 4-fluoro-7-nitrobenzofurazan derivatization. Biomed. Chromatogr 21: 273–278. 10.1002/bmc.750 17236239

[19] Himmelfarb J, McMenamin ME, Loseto G, Heinecke JW (2001) Myeloperoxidase-catalyzed 3-chlorotyrosine formation in dialysis patients. Free Radic Biol Med 31(10): 1163–1169. 11705694 10.1016/s0891-5849(01)00697-9

[20] Ischiropoulos H, Zhu L, Chen J, Tsai M, Martin JC, et al (1992) Peroxynitrite-mediated tyrosine nitration catalyzed by superoxide dismutase. Arch. Biochem Biophys 298: 431–437. 10.1016/0003-9861(92)90431-u 1416974

[21] Ohshima H, Friesen M, Brouet I, Bartsch H (1990) Nitrotyrosine as a new marker for endogenous nitrosation and nitration of proteins. Fund. Chem. Tox 28: 647–652. 10.1016/0278-6915(90)90173-k 2272563

[22] Radi R, Beckman JS, Bush KM, Freeman BA (1991) Peroxynitrite induced membrane lipid peroxidation: the cytotoxic potential of superoxide and nitric oxide. Arch. Biochem Biophys 288: 481–487. 10.1016/0003-9861(91)90224-7 1654835

[23] Gailit J, Colflesh D, Rabiner I, Simone J, Goligorsky MS (1993) Redistribution and dysfunction of integrins in cultured renal epithelial cells exposed to oxidative stress. Am J Physiol Renal Fluid Electrolyte Physiol 264: 149–157. 10.1152/ajprenal.1993.264.1.F149 8430825

[24] Graham A, Hogg N, Kalyanaraman B, O’Leary V, Darley-Usmar V, et al (1993) Peroxynitrite modification of lowdensity lipoprotein leads to recognition by the macrophage scavenger receptor. FEBS Lett 330: 181–185. 8365489 10.1016/0014-5793(93)80269-z

